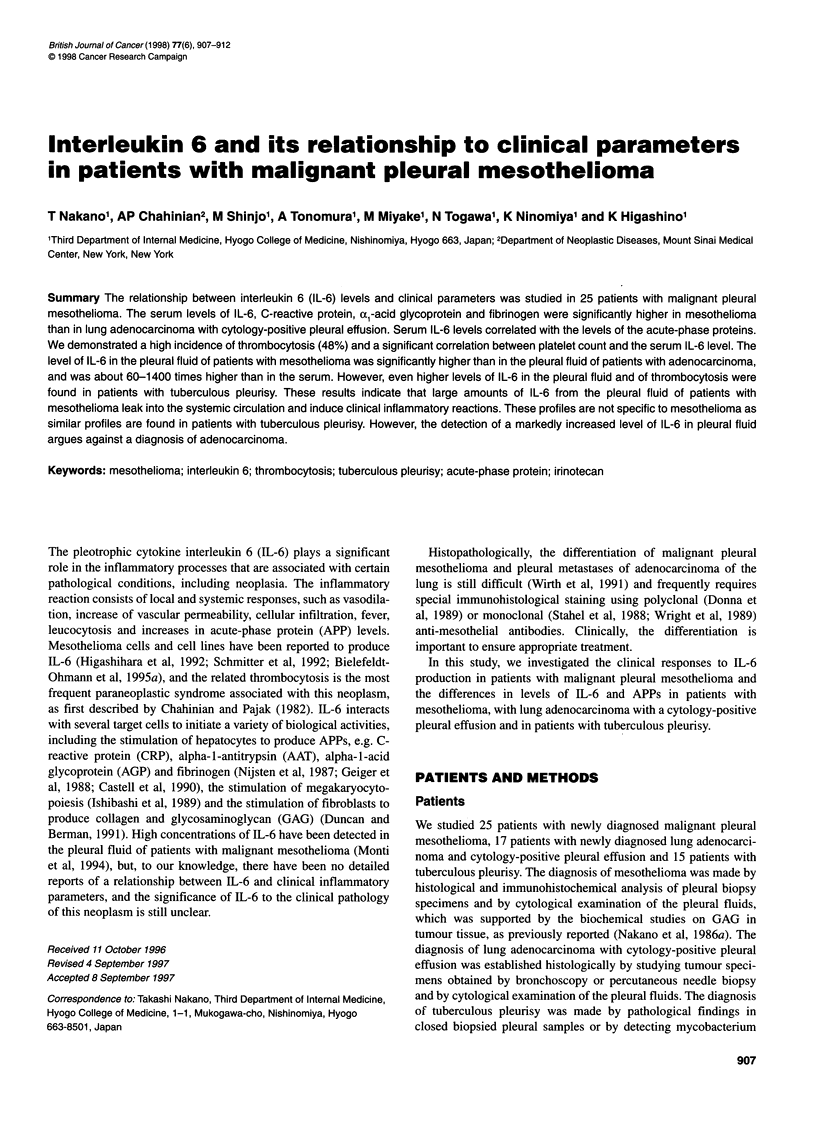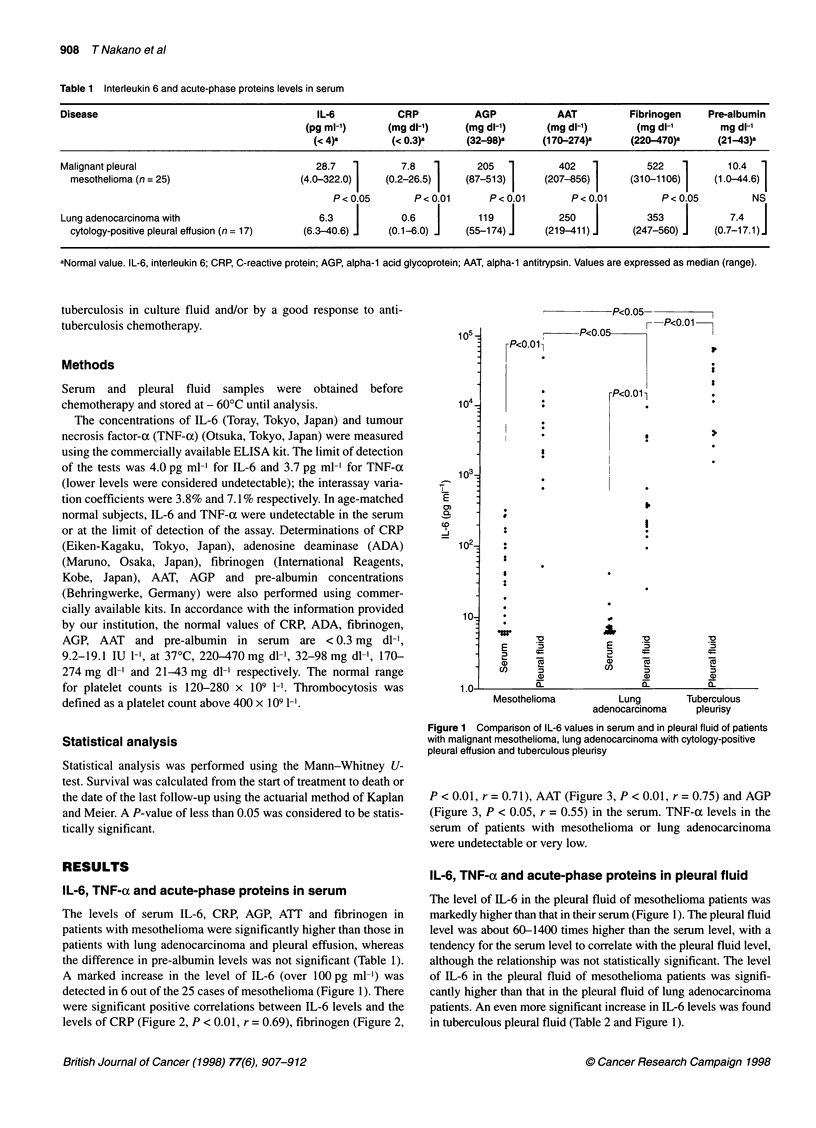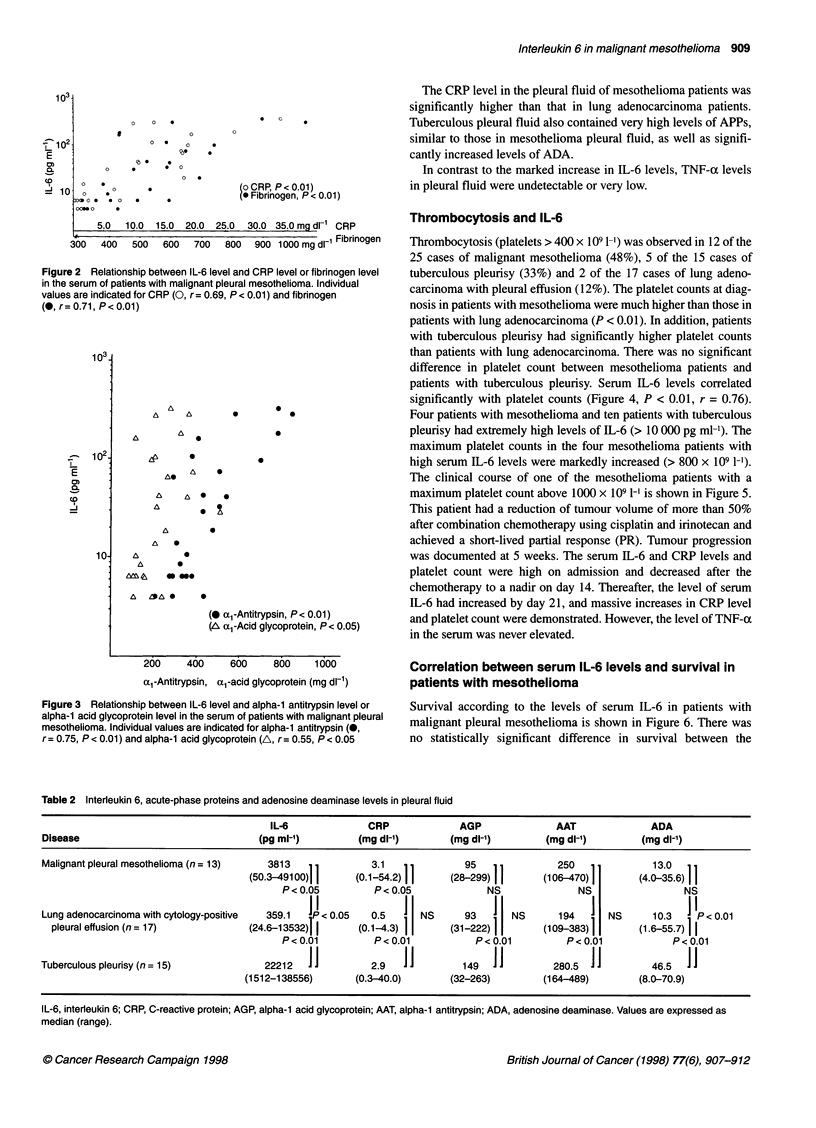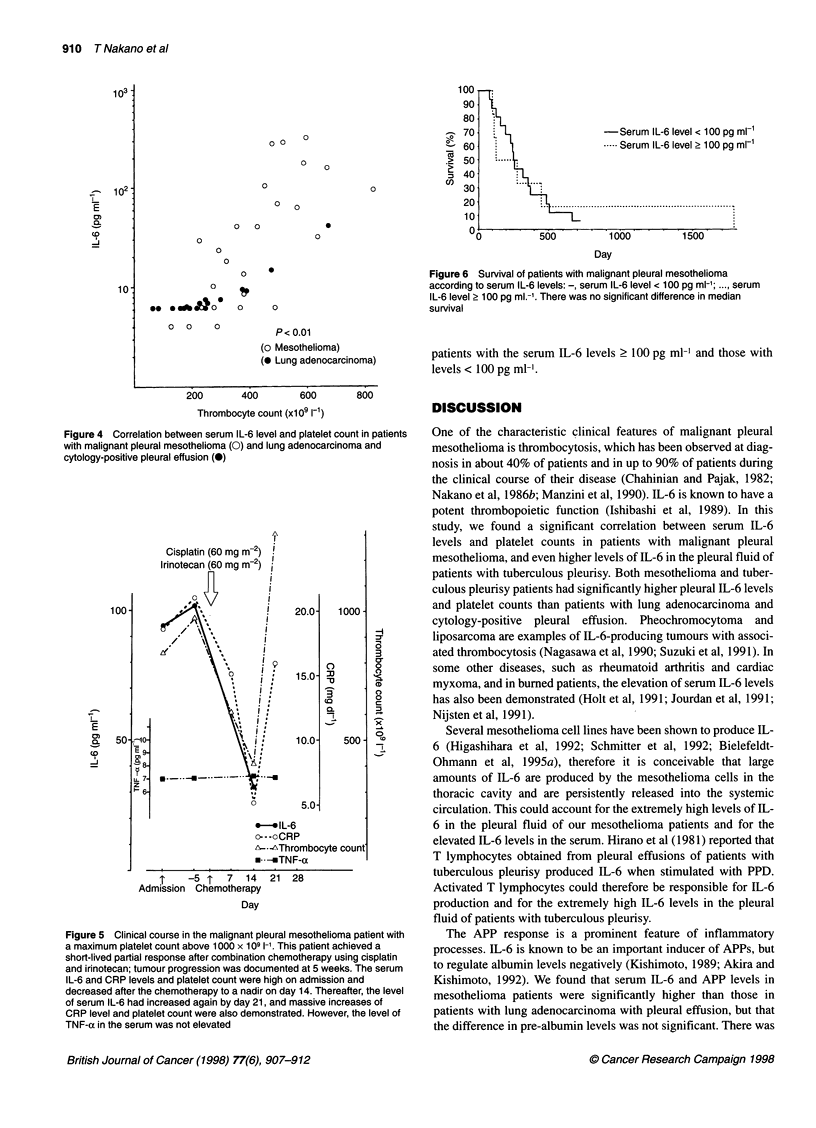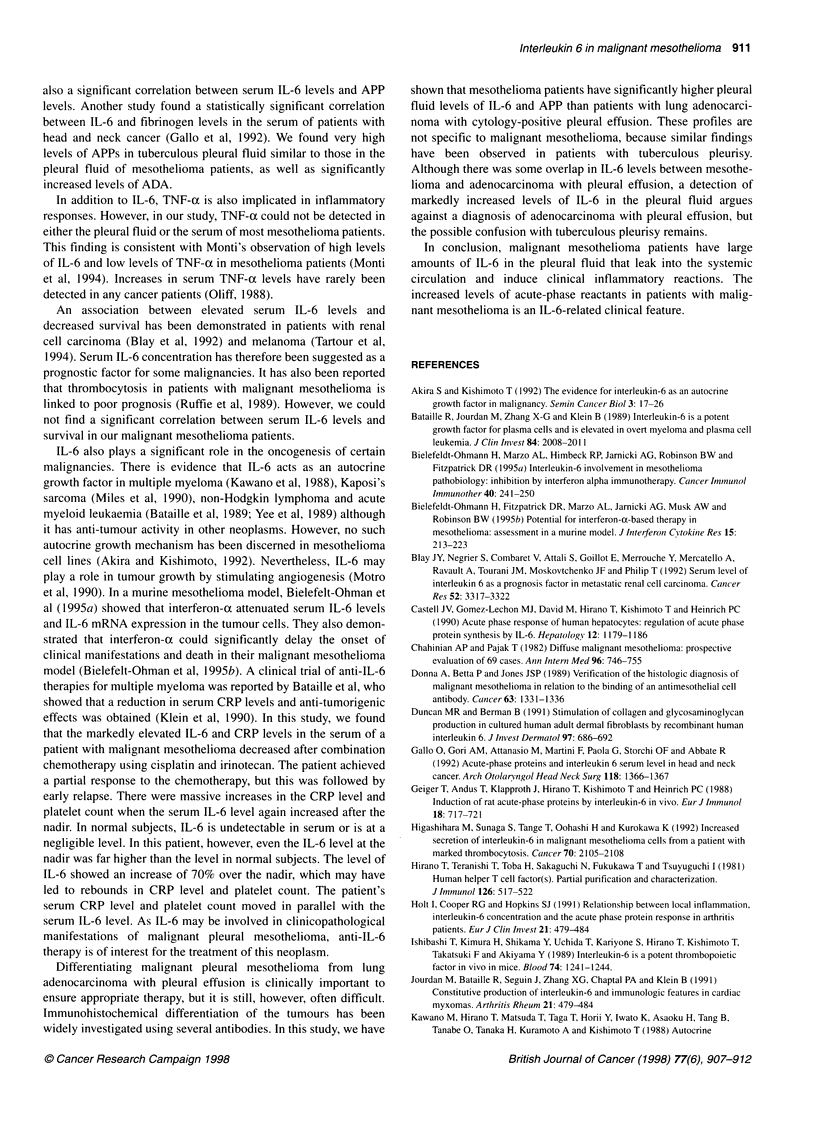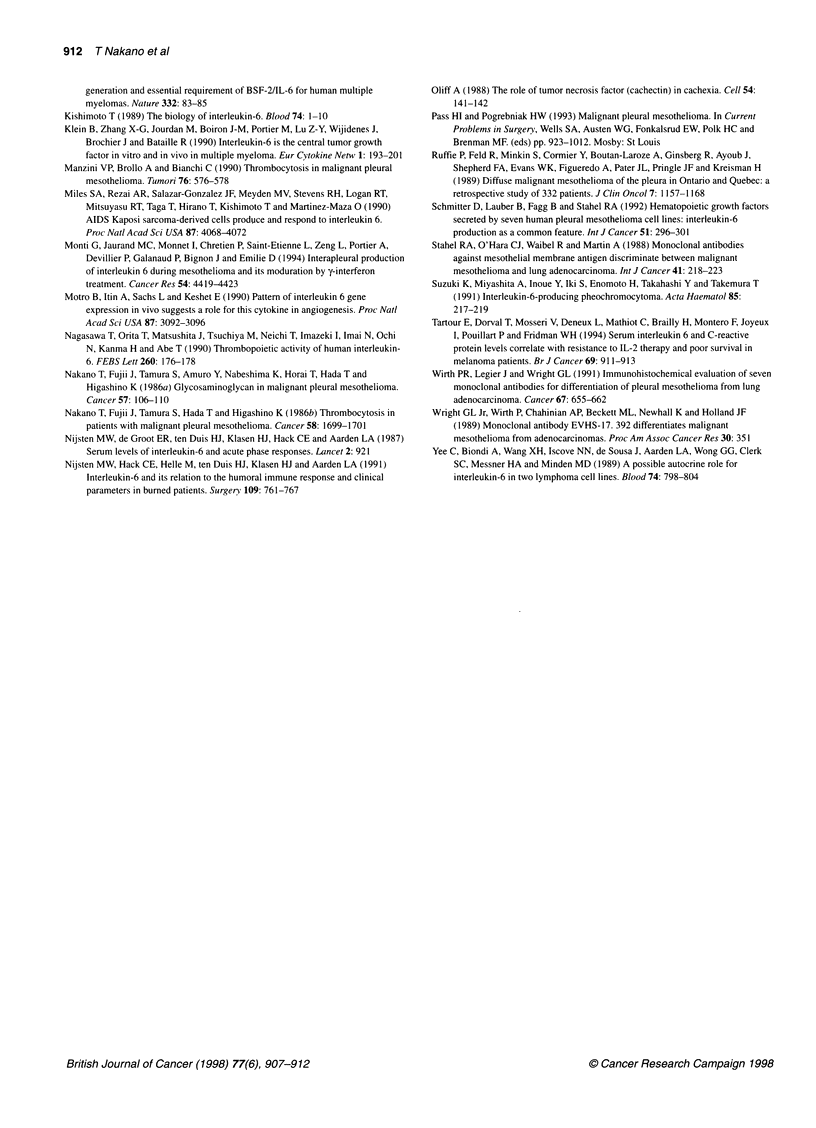# Interleukin 6 and its relationship to clinical parameters in patients with malignant pleural mesothelioma.

**DOI:** 10.1038/bjc.1998.150

**Published:** 1998-03

**Authors:** T. Nakano, A. P. Chahinian, M. Shinjo, A. Tonomura, M. Miyake, N. Togawa, K. Ninomiya, K. Higashino

**Affiliations:** Third Department of Internal Medicine, Hyogo College of Medicine, Nishinomiya, Japan.

## Abstract

The relationship between interleukin 6 (IL-6) levels and clinical parameters was studied in 25 patients with malignant pleural mesothelioma. The serum levels of IL-6, C-reactive protein, alpha1-acid glycoprotein and fibrinogen were significantly higher in mesothelioma than in lung adenocarcinoma with cytology-positive pleural effusion. Serum IL-6 levels correlated with the levels of the acute-phase proteins. We demonstrated a high incidence of thrombocytosis (48%) and a significant correlation between platelet count and the serum IL-6 level. The level of IL-6 in the pleural fluid of patients with mesothelioma was significantly higher than in the pleural fluid of patients with adenocarcinoma, and was about 60-1400 times higher than in the serum. However, even higher levels of IL-6 in the pleural fluid and of thrombocytosis were found in patients with tuberculous pleurisy. These results indicate that large amounts of IL-6 from the pleural fluid of patients with mesothelioma leak into the systemic circulation and induce clinical inflammatory reactions. These profiles are not specific to mesothelioma as similar profiles are found in patients with tuberculous pleurisy. However, the detection of a markedly increased level of IL-6 in pleural fluid argues against a diagnosis of adenocarcinoma.


British Journal of Cancer (1998) 77(6), 907–912
© 1998 Cancer Research Campaign

### Interleukin 6 and its relationship to clinical parameters in patients with malignant pleural mesothelioma

**T Nakano[1], AP Chahinian[2], M Shinjo[1], A Tonomura[1], M Miyake[1], N Togawa[1], K Ninomiya[1] and K Higashino[1]**

[1]Third Department of Internal Medicine, Hyogo College of Medicine, Nishinomiya, Hyogo 663, Japan; [2]Department of Neoplastic Diseases, Mount Sinai Medical Center, New York, New York

**Summary** The relationship between interleukin 6 (IL-6) levels and clinical parameters was studied in 25 patients with malignant pleural mesothelioma. The serum levels of IL-6, C-reactive protein, $\alpha_1$-acid glycoprotein and fibrinogen were significantly higher in mesothelioma than in lung adenocarcinoma with cytology-positive pleural effusion. Serum IL-6 levels correlated with the levels of the acute-phase proteins. We demonstrated a high incidence of thrombocytosis (48%) and a significant correlation between platelet count and the serum IL-6 level. The level of IL-6 in the pleural fluid of patients with mesothelioma was significantly higher than in the pleural fluid of patients with adenocarcinoma, and was about 60–1400 times higher than in the serum. However, even higher levels of IL-6 in the pleural fluid and of thrombocytosis were found in patients with tuberculous pleurisy. These results indicate that large amounts of IL-6 from the pleural fluid of patients with mesothelioma leak into the systemic circulation and induce clinical inflammatory reactions. These profiles are not specific to mesothelioma as similar profiles are found in patients with tuberculous pleurisy. However, the detection of a markedly increased level of IL-6 in pleural fluid argues against a diagnosis of adenocarcinoma.

**Keywords:** mesothelioma; interleukin 6; thrombocytosis; tuberculous pleurisy; acute-phase protein; irinotecan

The pleotrophic cytokine interleukin 6 (IL-6) plays a significant role in the inflammatory processes that are associated with certain pathological conditions, including neoplasia. The inflammatory reaction consists of local and systemic responses, such as vasodilation, increase of vascular permeability, cellular infiltration, fever, leucocytosis and increases in acute-phase protein (APP) levels. Mesothelioma cells and cell lines have been reported to produce IL-6 (Higashihara et al, 1992; Schmitter et al, 1992; Bielefeldt-Ohmann et al, 1995*a*), and the related thrombocytosis is the most frequent paraneoplastic syndrome associated with this neoplasm, as first described by Chahinian and Pajak (1982). IL-6 interacts with several target cells to initiate a variety of biological activities, including the stimulation of hepatocytes to produce APPs, e.g. C-reactive protein (CRP), alpha-1-antitrypsin (AAT), alpha-1-acid glycoprotein (AGP) and fibrinogen (Nijsten et al, 1987; Geiger et al, 1988; Castell et al, 1990), the stimulation of megakaryocytopoiesis (Ishibashi et al, 1989) and the stimulation of fibroblasts to produce collagen and glycosaminoglycan (GAG) (Duncan and Berman, 1991). High concentrations of IL-6 have been detected in the pleural fluid of patients with malignant mesothelioma (Monti et al, 1994), but, to our knowledge, there have been no detailed reports of a relationship between IL-6 and clinical inflammatory parameters, and the significance of IL-6 to the clinical pathology of this neoplasm is still unclear.

*Received 11 October 1996*
*Revised 4 September 1997*
*Accepted 8 September 1997*

*Correspondence to:* Takashi Nakano, Third Department of Internal Medicine, Hyogo College of Medicine, 1-1, Mukogawa-cho, Nishinomiya, Hyogo 663-8501, Japan

Histopathologically, the differentiation of malignant pleural mesothelioma and pleural metastases of adenocarcinoma of the lung is still difficult (Wirth et al, 1991) and frequently requires special immunohistological staining using polyclonal (Donna et al, 1989) or monoclonal (Stahel et al, 1988; Wright et al, 1989) anti-mesothelial antibodies. Clinically, the differentiation is important to ensure appropriate treatment.

In this study, we investigated the clinical responses to IL-6 production in patients with malignant pleural mesothelioma and the differences in levels of IL-6 and APPs in patients with mesothelioma, with lung adenocarcinoma with a cytology-positive pleural effusion and in patients with tuberculous pleurisy.

### PATIENTS AND METHODS

### Patients

We studied 25 patients with newly diagnosed malignant pleural mesothelioma, 17 patients with newly diagnosed lung adenocarcinoma and cytology-positive pleural effusion and 15 patients with tuberculous pleurisy. The diagnosis of mesothelioma was made by histological and immunohistochemical analysis of pleural biopsy specimens and by cytological examination of the pleural fluids, which was supported by the biochemical studies on GAG in tumour tissue, as previously reported (Nakano et al, 1986*a*). The diagnosis of lung adenocarcinoma with cytology-positive pleural effusion was established histologically by studying tumour specimens obtained by bronchoscopy or percutaneous needle biopsy and by cytological examination of the pleural fluids. The diagnosis of tuberculous pleurisy was made by pathological findings in closed biopsied pleural samples or by detecting mycobacterium

**Table 1** Interleukin 6 and acute-phase proteins levels in serum

| Disease | IL-6 (pg ml$^{-1}$) (< 4)[a] | CRP (mg dl$^{-1}$) (< 0.3)[a] | AGP (mg dl$^{-1}$) (32–98)[a] | AAT (mg dl$^{-1}$) (170–274)[a] | Fibrinogen (mg dl$^{-1}$ (220–470)[a] | Pre-albumin mg dl$^{-1}$ (21–43)[a] |
|---|---|---|---|---|---|---|
| Malignant pleural mesothelioma ($n$ = 25) | 28.7 (4.0–322.0) | 7.8 (0.2–26.5) | 205 (87–513) | 402 (207–856) | 522 (310–1106) | 10.4 (1.0–44.6) |
| | $P$ < 0.05 | $P$ < 0.01 | $P$ < 0.01 | $P$ < 0.01 | $P$ < 0.05 | NS |
| Lung adenocarcinoma with cytology-positive pleural effusion ($n$ = 17) | 6.3 (6.3–40.6) | 0.6 (0.1–6.0) | 119 (55–174) | 250 (219–411) | 353 (247–560) | 7.4 (0.7–17.1) |

[a]Normal value. IL-6, interleukin 6; CRP, C-reactive protein; AGP, alpha-1 acid glycoprotein; AAT, alpha-1 antitrypsin. Values are expressed as median (range).

tuberculosis in culture fluid and/or by a good response to anti-tuberculosis chemotherapy.

### Methods

Serum and pleural fluid samples were obtained before chemotherapy and stored at – 60°C until analysis.

The concentrations of IL-6 (Toray, Tokyo, Japan) and tumour necrosis factor-α (TNF-α) (Otsuka, Tokyo, Japan) were measured using the commercially available ELISA kit. The limit of detection of the tests was 4.0 pg ml$^{-1}$ for IL-6 and 3.7 pg ml$^{-1}$ for TNF-α (lower levels were considered undetectable); the interassay variation coefficients were 3.8% and 7.1% respectively. In age-matched normal subjects, IL-6 and TNF-α were undetectable in the serum or at the limit of detection of the assay. Determinations of CRP (Eiken-Kagaku, Tokyo, Japan), adenosine deaminase (ADA) (Maruno, Osaka, Japan), fibrinogen (International Reagents, Kobe, Japan), AAT, AGP and pre-albumin concentrations (Behringwerke, Germany) were also performed using commercially available kits. In accordance with the information provided by our institution, the normal values of CRP, ADA, fibrinogen, AGP, AAT and pre-albumin in serum are < 0.3 mg dl$^{-1}$, 9.2–19.1 IU l$^{-1}$, at 37°C, 220–470 mg dl$^{-1}$, 32–98 mg dl$^{-1}$, 170–274 mg dl$^{-1}$ and 21–43 mg dl$^{-1}$ respectively. The normal range for platelet counts is 120–280 × 10$^{9}$ l$^{-1}$. Thrombocytosis was defined as a platelet count above 400 × 10$^{9}$ l$^{-1}$.

### Statistical analysis

Statistical analysis was performed using the Mann–Whitney $U$-test. Survival was calculated from the start of treatment to death or the date of the last follow-up using the actuarial method of Kaplan and Meier. A $P$-value of less than 0.05 was considered to be statistically significant.

### RESULTS

### IL-6, TNF-α and acute-phase proteins in serum

The levels of serum IL-6, CRP, AGP, ATT and fibrinogen in patients with mesothelioma were significantly higher than those in patients with lung adenocarcinoma and pleural effusion, whereas the difference in pre-albumin levels was not significant (Table 1). A marked increase in the level of IL-6 (over 100 pg ml$^{-1}$) was detected in 6 out of the 25 cases of mesothelioma (Figure 1). There were significant positive correlations between IL-6 levels and the levels of CRP (Figure 2, $P$ < 0.01, $r$ = 0.69), fibrinogen (Figure 2,

**Figure 1** Comparison of IL-6 values in serum and in pleural fluid of patients with malignant mesothelioma, lung adenocarcinoma with cytology-positive pleural effusion and tuberculous pleurisy

$P$ < 0.01, $r$ = 0.71), AAT (Figure 3, $P$ < 0.01, $r$ = 0.75) and AGP (Figure 3, $P$ < 0.05, $r$ = 0.55) in the serum. TNF-α levels in the serum of patients with mesothelioma or lung adenocarcinoma were undetectable or very low.

### IL-6, TNF-α and acute-phase proteins in pleural fluid

The level of IL-6 in the pleural fluid of mesothelioma patients was markedly higher than that in their serum (Figure 1). The pleural fluid level was about 60–1400 times higher than the serum level, with a tendency for the serum level to correlate with the pleural fluid level, although the relationship was not statistically significant. The level of IL-6 in the pleural fluid of mesothelioma patients was significantly higher than that in the pleural fluid of lung adenocarcinoma patients. An even more significant increase in IL-6 levels was found in tuberculous pleural fluid (Table 2 and Figure 1).

 © Cancer Research Campaign 1998

**Figure 2** Relationship between IL-6 level and CRP level or fibrinogen level in the serum of patients with malignant pleural mesothelioma. Individual values are indicated for CRP (○, $r = 0.69$, $P < 0.01$) and fibrinogen (●, $r = 0.71$, $P < 0.01$)

**Figure 3** Relationship between IL-6 level and alpha-1 antitrypsin level or alpha-1 acid glycoprotein level in the serum of patients with malignant pleural mesothelioma. Individual values are indicated for alpha-1 antitrypsin (●, $r = 0.75$, $P < 0.01$) and alpha-1 acid glycoprotein (△, $r = 0.55$, $P < 0.05$

The CRP level in the pleural fluid of mesothelioma patients was significantly higher than that in lung adenocarcinoma patients. Tuberculous pleural fluid also contained very high levels of APPs, similar to those in mesothelioma pleural fluid, as well as significantly increased levels of ADA.

In contrast to the marked increase in IL-6 levels, TNF-α levels in pleural fluid were undetectable or very low.

### Thrombocytosis and IL-6

Thrombocytosis (platelets > 400 × $10^9$ $l^{-1}$) was observed in 12 of the 25 cases of malignant mesothelioma (48%), 5 of the 15 cases of tuberculous pleurisy (33%) and 2 of the 17 cases of lung adenocarcinoma with pleural effusion (12%). The platelet counts at diagnosis in patients with mesothelioma were much higher than those in patients with lung adenocarcinoma ($P < 0.01$). In addition, patients with tuberculous pleurisy had significantly higher platelet counts than patients with lung adenocarcinoma. There was no significant difference in platelet count between mesothelioma patients and patients with tuberculous pleurisy. Serum IL-6 levels correlated significantly with platelet counts (Figure 4, $P < 0.01$, $r = 0.76$). Four patients with mesothelioma and ten patients with tuberculous pleurisy had extremely high levels of IL-6 (> 10 000 pg $ml^{-1}$). The maximum platelet counts in the four mesothelioma patients with high serum IL-6 levels were markedly increased (> 800 × $10^9$ $l^{-1}$). The clinical course of one of the mesothelioma patients with a maximum platelet count above 1000 × $10^9$ $l^{-1}$ is shown in Figure 5. This patient had a reduction of tumour volume of more than 50% after combination chemotherapy using cisplatin and irinotecan and achieved a short-lived partial response (PR). Tumour progression was documented at 5 weeks. The serum IL-6 and CRP levels and platelet count were high on admission and decreased after the chemotherapy to a nadir on day 14. Thereafter, the level of serum IL-6 had increased by day 21, and massive increases in CRP level and platelet count were demonstrated. However, the level of TNF-α in the serum was never elevated.

### Correlation between serum IL-6 levels and survival in patients with mesothelioma

Survival according to the levels of serum IL-6 in patients with malignant pleural mesothelioma is shown in Figure 6. There was no statistically significant difference in survival between the

**Table 2** Interleukin 6, acute-phase proteins and adenosine deaminase levels in pleural fluid

| Disease | IL-6 (pg $ml^{-1}$) | CRP (mg $dl^{-1}$) | AGP (mg $dl^{-1}$) | AAT (mg $dl^{-1}$) | ADA (mg $dl^{-1}$) |
|---|---|---|---|---|---|
| Malignant pleural mesothelioma ($n$ = 13) | 3813 (50.3–49100) | 3.1 (0.1–54.2) | 95 (28–299) | 250 (106–470) | 13.0 (4.0–35.6) |
| | $P < 0.05$ | $P < 0.05$ | NS | NS | NS |
| Lung adenocarcinoma with cytology-positive pleural effusion ($n$ = 17) | 359.1 (24.6–13532) | 0.5 (0.1–4.3) | 93 (31–222) | 194 (109–383) | 10.3 (1.6–55.7) |
| | $P < 0.05$ | NS | NS | NS | $P < 0.01$ |
| | $P < 0.01$ | $P < 0.01$ | $P < 0.01$ | $P < 0.01$ | $P < 0.01$ |
| Tuberculous pleurisy ($n$ = 15) | 22212 (1512–138556) | 2.9 (0.3–40.0) | 149 (32–263) | 280.5 (164–489) | 46.5 (8.0–70.9) |

IL-6, interleukin 6; CRP, C-reactive protein; AGP, alpha-1 acid glycoprotein; AAT, alpha-1 antitrypsin; ADA, adenosine deaminase. Values are expressed as median (range).

**Figure 4** Correlation between serum IL-6 level and platelet count in patients with malignant pleural mesothelioma (○) and lung adenocarcinoma and cytology-positive pleural effusion (●)

**Figure 5** Clinical course in the malignant pleural mesothelioma patient with a maximum platelet count above 1000 × $10^9$ $l^{-1}$. This patient achieved a short-lived partial response after combination chemotherapy using cisplatin and irinotecan; tumour progression was documented at 5 weeks. The serum IL-6 and CRP levels and platelet count were high on admission and decreased after the chemotherapy to a nadir on day 14. Thereafter, the level of serum IL-6 had increased again by day 21, and massive increases of CRP level and platelet count were also demonstrated. However, the level of TNF-α in the serum was not elevated

**Figure 6** Survival of patients with malignant pleural mesothelioma according to serum IL-6 levels: –, serum IL-6 level < 100 pg $ml^{-1}$; ..., serum IL-6 level ≥ 100 pg $ml.^{-1}$. There was no significant difference in median survival patients with the serum IL-6 levels ≥ 100 pg $ml^{-1}$ and those with levels < 100 pg $ml^{-1}$.

### DISCUSSION

One of the characteristic clinical features of malignant pleural mesothelioma is thrombocytosis, which has been observed at diagnosis in about 40% of patients and in up to 90% of patients during the clinical course of their disease (Chahinian and Pajak, 1982; Nakano et al, 1986*b*; Manzini et al, 1990). IL-6 is known to have a potent thrombopoietic function (Ishibashi et al, 1989). In this study, we found a significant correlation between serum IL-6 levels and platelet counts in patients with malignant pleural mesothelioma, and even higher levels of IL-6 in the pleural fluid of patients with tuberculous pleurisy. Both mesothelioma and tuberculous pleurisy patients had significantly higher pleural IL-6 levels and platelet counts than patients with lung adenocarcinoma and cytology-positive pleural effusion. Pheochromocytoma and liposarcoma are examples of IL-6-producing tumours with associated thrombocytosis (Nagasawa et al, 1990; Suzuki et al, 1991). In some other diseases, such as rheumatoid arthritis and cardiac myxoma, and in burned patients, the elevation of serum IL-6 levels has also been demonstrated (Holt et al, 1991; Jourdan et al, 1991; Nijsten et al, 1991).

Several mesothelioma cell lines have been shown to produce IL-6 (Higashihara et al, 1992; Schmitter et al, 1992; Bielefeldt-Ohmann et al, 1995*a*), therefore it is conceivable that large amounts of IL-6 are produced by the mesothelioma cells in the thoracic cavity and are persistently released into the systemic circulation. This could account for the extremely high levels of IL-6 in the pleural fluid of our mesothelioma patients and for the elevated IL-6 levels in the serum. Hirano et al (1981) reported that T lymphocytes obtained from pleural effusions of patients with tuberculous pleurisy produced IL-6 when stimulated with PPD. Activated T lymphocytes could therefore be responsible for IL-6 production and for the extremely high IL-6 levels in the pleural fluid of patients with tuberculous pleurisy.

The APP response is a prominent feature of inflammatory processes. IL-6 is known to be an important inducer of APPs, but to regulate albumin levels negatively (Kishimoto, 1989; Akira and Kishimoto, 1992). We found that serum IL-6 and APP levels in mesothelioma patients were significantly higher than those in patients with lung adenocarcinoma with pleural effusion, but that the difference in pre-albumin levels was not significant. There was

© Cancer Research Campaign 1998

also a significant correlation between serum IL-6 levels and APP levels. Another study found a statistically significant correlation between IL-6 and fibrinogen levels in the serum of patients with head and neck cancer (Gallo et al, 1992). We found very high levels of APPs in tuberculous pleural fluid similar to those in the pleural fluid of mesothelioma patients, as well as significantly increased levels of ADA.

In addition to IL-6, TNF-α is also implicated in inflammatory responses. However, in our study, TNF-α could not be detected in either the pleural fluid or the serum of most mesothelioma patients. This finding is consistent with Monti's observation of high levels of IL-6 and low levels of TNF-α in mesothelioma patients (Monti et al, 1994). Increases in serum TNF-α levels have rarely been detected in any cancer patients (Oliff, 1988).

An association between elevated serum IL-6 levels and decreased survival has been demonstrated in patients with renal cell carcinoma (Blay et al, 1992) and melanoma (Tartour et al, 1994). Serum IL-6 concentration has therefore been suggested as a prognostic factor for some malignancies. It has also been reported that thrombocytosis in patients with malignant mesothelioma is linked to poor prognosis (Ruffie et al, 1989). However, we could not find a significant correlation between serum IL-6 levels and survival in our malignant mesothelioma patients.

IL-6 also plays a significant role in the oncogenesis of certain malignancies. There is evidence that IL-6 acts as an autocrine growth factor in multiple myeloma (Kawano et al, 1988), Kaposi's sarcoma (Miles et al, 1990), non-Hodgkin lymphoma and acute myeloid leukaemia (Bataille et al, 1989; Yee et al, 1989) although it has anti-tumour activity in other neoplasms. However, no such autocrine growth mechanism has been discerned in mesothelioma cell lines (Akira and Kishimoto, 1992). Nevertheless, IL-6 may play a role in tumour growth by stimulating angiogenesis (Motro et al, 1990). In a murine mesothelioma model, Bielefelt-Ohman et al (1995*a*) showed that interferon-α attenuated serum IL-6 levels and IL-6 mRNA expression in the tumour cells. They also demonstrated that interferon-α could significantly delay the onset of clinical manifestations and death in their malignant mesothelioma model (Bielefelt-Ohman et al, 1995*b*). A clinical trial of anti-IL-6 therapies for multiple myeloma was reported by Bataille et al, who showed that a reduction in serum CRP levels and anti-tumorigenic effects was obtained (Klein et al, 1990). In this study, we found that the markedly elevated IL-6 and CRP levels in the serum of a patient with malignant mesothelioma decreased after combination chemotherapy using cisplatin and irinotecan. The patient achieved a partial response to the chemotherapy, but this was followed by early relapse. There were massive increases in the CRP level and platelet count when the serum IL-6 level again increased after the nadir. In normal subjects, IL-6 is undetectable in serum or is at a negligible level. In this patient, however, even the IL-6 level at the nadir was far higher than the level in normal subjects. The level of IL-6 showed an increase of 70% over the nadir, which may have led to rebounds in CRP level and platelet count. The patient's serum CRP level and platelet count moved in parallel with the serum IL-6 level. As IL-6 may be involved in clinicopathological manifestations of malignant pleural mesothelioma, anti-IL-6 therapy is of interest for the treatment of this neoplasm.

Differentiating malignant pleural mesothelioma from lung adenocarcinoma with pleural effusion is clinically important to ensure appropriate therapy, but it is still, however, often difficult. Immunohistochemical differentiation of the tumours has been widely investigated using several antibodies. In this study, we have shown that mesothelioma patients have significantly higher pleural fluid levels of IL-6 and APP than patients with lung adenocarcinoma with cytology-positive pleural effusion. These profiles are not specific to malignant mesothelioma, because similar findings have been observed in patients with tuberculous pleurisy. Although there was some overlap in IL-6 levels between mesothelioma and adenocarcinoma with pleural effusion, a detection of markedly increased levels of IL-6 in the pleural fluid argues against a diagnosis of adenocarcinoma with pleural effusion, but the possible confusion with tuberculous pleurisy remains.

In conclusion, malignant mesothelioma patients have large amounts of IL-6 in the pleural fluid that leak into the systemic circulation and induce clinical inflammatory reactions. The increased levels of acute-phase reactants in patients with malignant mesothelioma is an IL-6-related clinical feature.

### REFERENCES

Akira S and Kishimoto T (1992) The evidence for interleukin-6 as an autocrine growth factor in malignancy. *Semin Cancer Biol* **3**: 17–26

Bataille R, Jourdan M, Zhang X-G and Klein B (1989) Interleukin-6 is a potent growth factor for plasma cells and is elevated in overt myeloma and plasma cell leukemia. *J Clin Invest* **84**: 2008–2011

Bielefeldt-Ohmann H, Marzo AL, Himbeck RP, Jarnicki AG, Robinson BW and Fitzpatrick DR (1995*a*) Interleukin-6 involvement in mesothelioma pathobiology: inhibition by interferon alpha immunotherapy. *Cancer Immunol Immunother* **40**: 241–250

Bielefeldt-Ohmann H, Fitzpatrick DR, Marzo AL, Jarnicki AG, Musk AW and Robinson BW (1995*b*) Potential for interferon-α-based therapy in mesothelioma: assessment in a murine model. *J Interferon Cytokine Res* **15**: 213–223

Blay JY, Negrier S, Combaret V, Attali S, Goillot E, Merrouche Y, Mercatello A, Ravault A, Tourani JM, Moskovtchenko JF and Philip T (1992) Serum level of interleukin 6 as a prognosis factor in metastatic renal cell carcinoma. *Cancer Res* **52**: 3317–3322

Castell JV, Gomez-Lechon MJ, David M, Hirano T, Kishimoto T and Heinrich PC (1990) Acute phase response of human hepatocytes: regulation of acute phase protein synthesis by IL-6. *Hepatology* **12**: 1179–1186

Chahinian AP and Pajak T (1982) Diffuse malignant mesothelioma: prospective evaluation of 69 cases. *Ann Intern Med* **96**: 746–755

Donna A, Betta P and Jones JSP (1989) Verification of the histologic diagnosis of malignant mesothelioma in relation to the binding of an antimesothelial cell antibody. *Cancer* **63**: 1331–1336

Duncan MR and Berman B (1991) Stimulation of collagen and glycosaminoglycan production in cultured human adult dermal fibroblasts by recombinant human interleukin 6. *J Invest Dermatol* **97**: 686–692

Gallo O, Gori AM, Attanasio M, Martini F, Paola G, Storchi OF and Abbate R (1992) Acute-phase proteins and interleukin 6 serum level in head and neck cancer. *Arch Otolaryngol Head Neck Surg* **118**: 1366–1367

Geiger T, Andus T, Klapproth J, Hirano T, Kishimoto T and Heinrich PC (1988) Induction of rat acute-phase proteins by interleukin-6 in vivo. *Eur J Immunol* **18**: 717–721

Higashihara M, Sunaga S, Tange T, Oohashi H and Kurokawa K (1992) Increased secretion of interleukin-6 in malignant mesothelioma cells from a patient with marked thrombocytosis. *Cancer* **70**: 2105–2108

Hirano T, Teranishi T, Toba H, Sakaguchi N, Fukukawa T and Tsuyuguchi I (1981) Human helper T cell factor(s). Partial purification and characterization. *J Immunol* **126**: 517–522

Holt I, Cooper RG and Hopkins SJ (1991) Relationship between local inflammation, interleukin-6 concentration and the acute phase protein response in arthritis patients. *Eur J Clin Invest* **21**: 479–484

Ishibashi T, Kimura H, Shikama Y, Uchida T, Kariyone S, Hirano T, Kishimoto T, Takatsuki F and Akiyama Y (1989) Interleukin-6 is a potent thrombopoietic factor in vivo in mice. *Blood* **74**: 1241–1244.

Jourdan M, Bataille R, Seguin J, Zhang XG, Chaptal PA and Klein B (1991) Constitutive production of interleukin-6 and immunologic features in cardiac myxomas. *Arthritis Rheum* **21**: 479–484

Kawano M, Hirano T, Matsuda T, Taga T, Horii Y, Iwato K, Asaoku H, Tang B, Tanabe O, Tanaka H, Kuramoto A and Kishimoto T (1988) Autocrine

© Cancer Research Campaign 1998

generation and essential requirement of BSF-2/IL-6 for human multiple myelomas. *Nature* **332**: 83–85

Kishimoto T (1989) The biology of interleukin-6. *Blood* **74**: 1–10

Klein B, Zhang X-G, Jourdan M, Boiron J-M, Portier M, Lu Z-Y, Wijidenes J, Brochier J and Bataille R (1990) Interleukin-6 is the central tumor growth factor in vitro and in vivo in multiple myeloma. *Eur Cytokine Netw* **1**: 193–201

Manzini VP, Brollo A and Bianchi C (1990) Thrombocytosis in malignant pleural mesothelioma. *Tumori* **76**: 576–578

Miles SA, Rezai AR, Salazar-Gonzalez JF, Meyden MV, Stevens RH, Logan RT, Mitsuyasu RT, Taga T, Hirano T, Kishimoto T and Martinez-Maza O (1990) AIDS Kaposi sarcoma-derived cells produce and respond to interleukin 6. *Proc Natl Acad Sci USA* **87**: 4068–4072

Monti G, Jaurand MC, Monnet I, Chretien P, Saint-Etienne L, Zeng L, Portier A, Devillier P, Galanaud P, Bignon J and Emilie D (1994) Interapleural production of interleukin 6 during mesothelioma and its moduration by γ-interferon treatment. *Cancer Res* **54**: 4419–4423

Motro B, Itin A, Sachs L and Keshet E (1990) Pattern of interleukin 6 gene expression in vivo suggests a role for this cytokine in angiogenesis. *Proc Natl Acad Sci USA* **87**: 3092–3096

Nagasawa T, Orita T, Matsushita J, Tsuchiya M, Neichi T, Imazeki I, Imai N, Ochi N, Kanma H and Abe T (1990) Thrombopoietic activity of human interleukin-6. *FEBS Lett* **260**: 176–178

Nakano T, Fujii J, Tamura S, Amuro Y, Nabeshima K, Horai T, Hada T and Higashino K (1986*a*) Glycosaminoglycan in malignant pleural mesothelioma. *Cancer* **57**: 106–110

Nakano T, Fujii J, Tamura S, Hada T and Higashino K (1986*b*) Thrombocytosis in patients with malignant pleural mesothelioma. *Cancer* **58**: 1699–1701

Nijsten MW, de Groot ER, ten Duis HJ, Klasen HJ, Hack CE and Aarden LA (1987) Serum levels of interleukin-6 and acute phase responses. *Lancet* **2**: 921

Nijsten MW, Hack CE, Helle M, ten Duis HJ, Klasen HJ and Aarden LA (1991) Interleukin-6 and its relation to the humoral immune response and clinical parameters in burned patients. *Surgery* **109**: 761–767

Oliff A (1988) The role of tumor necrosis factor (cachectin) in cachexia. *Cell* **54**: 141–142

Pass HI and Pogrebniak HW (1993) Malignant pleural mesothelioma. In *Current Problems in Surgery*, Wells SA, Austen WG, Fonkalsrud EW, Polk HC and Brenman MF. (eds) pp. 923–1012. Mosby: St Louis

Ruffie P, Feld R, Minkin S, Cormier Y, Boutan-Laroze A, Ginsberg R, Ayoub J, Shepherd FA, Evans WK, Figueredo A, Pater JL, Pringle JF and Kreisman H (1989) Diffuse malignant mesothelioma of the pleura in Ontario and Quebec: a retrospective study of 332 patients. *J Clin Oncol* **7**: 1157–1168

Schmitter D, Lauber B, Fagg B and Stahel RA (1992) Hematopoietic growth factors secreted by seven human pleural mesothelioma cell lines: interleukin-6 production as a common feature. *Int J Cancer* **51**: 296–301

Stahel RA, O'Hara CJ, Waibel R and Martin A (1988) Monoclonal antibodies against mesothelial membrane antigen discriminate between malignant mesothelioma and lung adenocarcinoma. *Int J Cancer* **41**: 218–223

Suzuki K, Miyashita A, Inoue Y, Iki S, Enomoto H, Takahashi Y and Takemura T (1991) Interleukin-6-producing pheochromocytoma. *Acta Haematol* **85**: 217–219

Tartour E, Dorval T, Mosseri V, Deneux L, Mathiot C, Brailly H, Montero F, Joyeux I, Pouillart P and Fridman WH (1994) Serum interleukin 6 and C-reactive protein levels correlate with resistance to IL-2 therapy and poor survival in melanoma patients. *Br J Cancer* **69**: 911–913

Wirth PR, Legier J and Wright GL (1991) Immunohistochemical evaluation of seven monoclonal antibodies for differentiation of pleural mesothelioma from lung adenocarcinoma. *Cancer* **67**: 655–662

Wright GL Jr, Wirth P, Chahinian AP, Beckett ML, Newhall K and Holland JF (1989) Monoclonal antibody EVHS-17. 392 differentiates malignant mesothelioma from adenocarcinomas. *Proc Am Assoc Cancer Res* **30**: 351

Yee C, Biondi A, Wang XH, Iscove NN, de Sousa J, Aarden LA, Wong GG, Clerk SC, Messner HA and Minden MD (1989) A possible autocrine role for interleukin-6 in two lymphoma cell lines. *Blood* **74**: 798–804

*© Cancer Research Campaign 1998*